# Understanding clinical reasoning in osteopathy: a qualitative research approach

**DOI:** 10.1186/s12998-016-0087-x

**Published:** 2016-03-08

**Authors:** Sandra Grace, Paul Orrock, Brett Vaughan, Raymond Blaich, Rosanne Coutts

**Affiliations:** School of Health & Human Sciences, Southern Cross University, PO Box 157, Lismore, NSW 2480 Australia; College of Health & Biomedicine, Victoria University, 301 Flinders Lane, Melbourne, Australia

## Abstract

**Background:**

Clinical reasoning has been described as a process that draws heavily on the knowledge, skills and attributes that are particular to each health profession. However, the clinical reasoning processes of practitioners of different disciplines demonstrate many similarities, including hypothesis generation and reflective practice. The aim of this study was to understand clinical reasoning in osteopathy from the perspective of osteopathic clinical educators and the extent to which it was similar or different from clinical reasoning in other health professions.

**Methods:**

This study was informed by constructivist grounded theory. Participants were clinical educators in osteopathic teaching institutions in Australia, New Zealand and the UK. Focus groups and written critical reflections provided a rich data set. Data were analysed using constant comparison to develop inductive categories.

**Results:**

According to participants, clinical reasoning in osteopathy is different from clinical reasoning in other health professions. Osteopaths use a two-phase approach: an initial biomedical screen for serious pathology, followed by use of osteopathic reasoning models that are based on the relationship between structure and function in the human body. Clinical reasoning in osteopathy was also described as occurring in a number of contexts (e.g. patient, practitioner and community) and drawing on a range of metaskills (e.g. hypothesis generation and reflexivity) that have been described in other health professions.

**Conclusions:**

The use of diagnostic reasoning models that are based on the relationship between structure and function in the human body differentiated clinical reasoning in osteopathy. These models were not used to name a medical condition but rather to guide the selection of treatment approaches. If confirmed by further research that clinical reasoning in osteopathy is distinct from clinical reasoning in other health professions, then osteopaths may have a unique perspective to bring to multidisciplinary decision-making and potentially enhance the quality of patient care.

Where commonalities exist in the clinical reasoning processes of osteopathy and other health professions, shared learning opportunities may be available, including the exchange of scaffolded clinical reasoning exercises and assessment practices among health disciplines.

## Background

Current health reforms have promoted team-based care, new scopes of practice, new health care roles and sharing of professional information [1, 2] which can cross professional boundaries and challenge our notions of professional identity. Professional boundaries are based on sets of competencies and unifying philosophies, and codified by formal education [3]. Professional identities, created through monopolising a body of knowledge and skills [4], are challenged by practitioners who practise within advanced and extended scopes, as occurs when podiatrists perform minor surgical procedures, or when nurse practitioners prescribe medication. It has been suggested that knowledge in a particular area of discipline-specific expertise may be less important for future health practitioners than generic skills like teamwork, and sound and reasoned judgement [5].

Clinical reasoning broadly refers to the ‘thinking and decision-making processes associated with clinical practice’ [6]. Traditional models of clinical reasoning referred to a cognitive practitioner-centred process whereby practitioners gather information about their patients, synthesise that information and develop treatment and management plans [7, 8]. Cognitive models tend to be very structured in their descriptions of how reasoning takes place. They also tend to be have little consideration for the patient’s contribution to the reasoning process, despite practitioners’ heavy reliance on context in the reasoning process [9].

How reasoning occurs for practitioners of varying experience (i.e. novice or experienced) has been a strong focus in the literature on clinical reasoning. Analytical or hypothetico-deductive reasoning was initially associated solely with the reasoning processes of novice practitioners. Expertise, on the other hand, was associated with intuitive reasoning or pattern recognition. Hallmarks of expertise included being able to identify and synthesise important clinical information, particularly for complaints that are ill-defined or complex [10], using metacognitive skills [11, 12], having life and clinical experience [13], and having insight into one’s own reasoning processes [14]. It was previously thought that experienced practitioners, when presented with cases that did not fit into recognisable patterns, reverted to a hypothetico-deductive approach. However, it appears that these two approaches to problem solving are fluid and overlapping, practitioners dynamically moving between both, depending on the situation [15]. Peterson [16] demonstrated that radiology students who performed well on a radiology film-reading examination exhibited similar reasoning characteristics to those practitioners with many years’ experience. Moreover, it appears that no two practitioners will follow the same reasoning process even if the presenting complaint is exactly the same and the same diagnosis is reached [17]. The dual processing theory of clinical reasoning [18] has been proposed to describe practitioners’ use of both non-analytical processes (intuition or pattern recognition) and analytical ones (hypthetico-deductive) in clinical reasoning. This theory recognises the high level of interaction between the two processes.

Another approach to clinical reasoning in the health professions that has been described by Hamm [19] was based on the cognitive continuum theory proposed by Hammond [20]. In fact, it has been argued that that dual processing model should be replaced by cognitive continuum theory as the predominant theory to explain clinical reasoning [21]. Cognitive continuum theory is underpinned by the two concepts of cognition and task properties. Within cognition is a continuum from analysis (slow, deliberate task/data processing, high level of confidence in outcome) to intuition (fast task/data processing, low level of confidence in outcome). The central part of this continuum is referred to as quasirationality: a combination of analysis and intuition. Most clinical reasoning is thought to lie within quasirationality [21]. Task properties can be described as well- and ill-structured: well-structured tasks require time to process and resolve, whereas ill-structured tasks require a quick resolution with little time devoted to contrasting different outcomes. Changing a task is likely to influence the point along the cognition continuum which describes a clinician’s reasoning. It is the specific features of a task and mode of processing that Custers [21] suggests is an under-developed area in the clinical reasoning literature.

Sociocultural or interpretive models of clinical reasoning prioritise the centrality of the patient in clinical reasoning and emphasise the collaborative and interactive nature of the process [22, 23]. Edwards et al. [12] found that physiotherapists moved between reasoning about the patients’ physical complaints (hypothetico-deductive or pattern recognition) and engaging with the patient or their carer (narrative reasoning) to understand the impact of the complaint. Higgs and Jones [24] described a number of contexts of clinical reasoning that included all aspects of the health encounter including patients, practitioners’ interpersonal skills and personal values, teams, the workplace, and the local and global health systems where interactions take place (see Table 1). In this interpretation, collaborative reasoning could include interactions between patients and practitioners, and/or practitioners and other practitioners to co-create decisions about health care.

**Table 1:** Key contexts and metaskills of clinical reasoning (adapted from Higgs and Jones [24])

Contexts	Practitioner	• Practice knowledge • Practice experience • Values and beliefs • Own professional practice
	Patient	• Values and beliefs • Health and illness experiences • Knowledge and experience of the health discipline • Knowledge and experience of other health disciplines and the health care system
	Community	• Patient’s family and friends • The health discipline • Local and global health systems • Workplace
Metaskills		• Reflexivity • Metacognition • Emotional intelligence • Analytic skills and pattern recognition • Knowledge generation • Practice-model authenticity • Ability to derive knowledge and practice wisdom from reasoning and practice • Use of critical, creative conversation to make decisions

### Clinical reasoning in specific health professions

Clinical reasoning has been described as a process that draws heavily on the professional learning, craft knowledge and intuition [25] that are particular to each health profession [26, 27]. Fleming and Mattingly [28] argued that the hypothetical deductive reasoning that emerged from the medical problem-solving tradition was too narrow to encompass ‘the myriad ways in which health professionals devise solutions for clients’ needs.’ In an earlier work Fleming [29] compared medical and occupational therapy clinical reasoning and found both similarities that arose from using hypothetical reasoning and differences that arose from the ‘particular focus, goals, and tasks of the two professions and the nature of the practice in those arenas.’

Metaskills can be broadly defined as higher-order skills that enable effective use of pre-existing skills (see Table 1). The use of metaskills has been identified as part of the clinical reasoning process in many health professions [30] Metaskills include: knowledge/hypothesis generation when differential diagnoses are formulated from information collected from or about patients [31, 32]; reflexivity that requires a combination of reflection on one’s own clinical practice and identification of future learning needs; the ability to derive knowledge and practice wisdom from reasoning and practice; the use of critical, creative conversation to make decisions; and the ability to locate reasoning as behaviours and strategies within chosen practice models, each with an inherent philosophy of practice [24]. This last metaskill is also referred to as ‘practice model authenticity’ and suggests that clinical reasoning is likely to differ according to the underpinning philosophy and principles of a particular health profession.

### Clinical reasoning in manual therapies

Manual therapies includes corrective exercise, chiropractic, osteopathy, physiotherapy, massage therapy, and muscle training [33]. The World Confederation for Physical Therapy [34] describes physical therapy as providing ‘services that develop, maintain and restore people’s maximum movement and functional ability. They can help people at any stage of life, when movement and function are threatened by ageing, injury, diseases, disorders, conditions or environmental factors … Physical therapists help people maximise their quality of life, looking at physical, psychological, emotional and social wellbeing. They work in the health spheres of promotion, prevention, treatment/intervention, habilitation and rehabilitation.’ Such a description could encompass the practices of many manual therapy professions.

A number of studies of the clinical reasoning practices in specific manual therapy professions are reported in the literature. For example, Ajjawi [35] found that hypothetico-deductive reasoning was used as the predominant strategy in physiotherapy. Components of clinical reasoning that were originally described by Elstein in medicine [8], namely cue acquisition, hypothesis generation, cue interpretation and hypothesis evaluation, were still evident in physiotherapy although contemporary clinical reasoning took in case management as well as diagnosis [35]. Manual therapists have also been reported as embracing the biopsychosocial model, becoming more holistic in their approach, and having more focus on active management and patient participation [36].

Jones and Rivett [37] set out to develop the clinical reasoning skills of manual therapists using a series of cases with commentary. These authors argued that ‘the original professional training of the manual therapists, whether it be in physiotherapy, chiropractic, osteopathy, medicine or another profession, is not important because the clinical reasoning process is universal’ [37].

### Clinical reasoning in osteopathy

Sociocultural and interpretive approaches to clinical reasoning may be particularly well suited to professions like osteopathy that are based on different philosophical foundations from medicine and that have traditionally emphasised holism, patient-centredness, and a wellness model of care [38]. The personal values of the practitioner and their professional belief system will influence their clinical reasoning [24]. The practice of osteopathy is reportedly founded on a set of principles [39], although it is unclear how these principles influence the clinical reasoning employed by osteopaths. A number of osteopathic reasoning models based on the relationship between structure and function in the human body have been developed to facilitate interpretation of subjective information collected from patients and objective data from diagnostic testing [40]. These structure-function relationships include biomechanical, psychosocial, neurological, nutritional, respiratory-circulatory and energy-expenditure models (see Table 2). The structure-function relationships are used to prioritise specific osteopathic treatment approaches and the order in which they are applied. However, despite the suggested inclusion of structure-function relationship models in osteopathic curricula and their use in practice, there is little in the literature that explores their application in osteopathic clinical reasoning. In fact, clinical reasoning in osteopathy has only recently been described in the literature. According to Thomson et al. [41], clinical reasoning for experienced practitioners lies along a continuum of practice from technical rationality (a practitioner-centred, physical, biomedical and biomechanical approach) to professional artistry (a patient-centred, biopsychosocial approach) - a continuum that encompasses hypothetico-deductive reasoning, pattern recognition and narrative reasoning (collaborative dialogue between the patient and practitioner to co-produce treatments). In the current climate of health care reform, it is timely that the osteopathic profession explores its professional identity and the clinical reasoning that underpins it [42].

**Table 2:** Osteopathic diagnostic models

Biomedical	Consideration of signs and symptoms in the context of defined diseases and need for referral for further medical assessment and management (red flags).
Biomechanical	Assessment of the health of the musculoskeletal system, including how the structure (posture) and function are integrated.
Respiratory/circulatory	Examination of the respiratory mechanism, ensuring that breathing function is optimal. Assessment of all tissues of the body for full blood supply and drainage, and of the structural and functional relationship between the two systems.
Neurological	Assessment of function in the central, peripheral and autonomic nervous systems, and the relationship of those systems to all tissues of the body.
Nutritional	Foundational dietary analysis for signs of deficiency or suboptimal nutritional status.
Behavioural	Consideration of the psychosocial factors influencing health, including relational, occupational and financial, and the need for multidisciplinary care.
Energy expenditure	Assessment of optimal energy utilisation, and consideration of issues that may affect the healing process (e.g. relatively minor mechanical or immune dysfunctions).

The purpose of this study was to explore osteopathic clinical educators’ understanding of clinical reasoning in the broader context of clinical reasoning in the health professions, in order to ascertain commonalities and differences. Clinical educators were selected for this study because of their pivotal role in developing and assessing clinical reasoning skills in students. Osteopathic educators regularly engage with the design, conduct and marking of clinical assessments that purport to assess clinical reasoning in osteopathy. They are well positioned to reflect on their understanding of clinical reasoning and ways in which those understandings were shaped by their experiences as educators and practitioners. They are likely to have developed an understanding of the processes of clinical reasoning and of strategies to scaffold development of clinical reasoning in osteopathy students. The present study draws on the perspectives of clinical educators from osteopathic programs in Australia, New Zealand and the UK. Findings of this study will contribute to our understanding of clinical reasoning in osteopathy and will inform our understanding of the contribution of osteopathic clinical reasoning to patient health care and to curriculum development.

## Methods

This research drew on elements of constructivist grounded theory. We assumed that clinical reasoning is created by practitioners as they interact with and interpret objects in the world [43]. In the present study ‘objects’ include patients’ signs and symptoms, current literature, practitioners’ previous experiences and colleagues’ opinions. According to Charmaz [44], researcher and participant construct a shared reality through an iterative process of data collection and analysis. Researchers’ perspectives formed part of the analysis in the present study. The research team comprised four osteopaths and one exercise physiologist, the latter also facilitated the focus groups. The strategy of using a facilitator from another discipline was to bring a perspective from another health discipline to the data analysis.

A purposive sample of participants from three Australian universities and two international osteopathic programs (one university in New Zealand and one college in the United Kingdom) were invited to contribute because of strong working relationships between the institutions. Four institutions accepted the invitation: four participants from Southern Cross University (SCU) (Australia), two participants from each of Victoria University (VU) (Australia), and Unitec (New Zealand), and one participant from the British School of Osteopathy (BSO) (United Kingdom), providing an appropriate cross-section of the profession for a preliminary investigation. This cross-section of participants is also relevant because of the capacity for osteopaths to move between these three countries to practise. Table 3 provides demographic data of the study participants. The study was approved by the SCU Human Research Ethics Committee (Approval number ECN-12-232). Data collection and analysis took place during 2013 and 2014.

**Table 3:** Participant demographics

	Gender	Age (years)	Institution	Years in practice	Years of clinical supervision
1^a^	M	51-60	SCU	25	22
2^a^	M	41-50	SCU	20	16
3^a^	F	51-60	SCU	32	30
4	F	51-60	SCU	25	22
5	F	41-50	VU	13	8
6^a^	M	41-50	VU	13	10
7	F	41-50	Unitec	21	20
8	M	41-50	Unitec	16	14
9	M	41-50	BSO	20	18
10^a^	F	51-60	SCU	12	5

^a^Members of the research team

Note: 10 was the facilitator; an exercise physiologist, not a registered osteopath

Osteopathic educators were invited to participate in two data collection activities in which participants individually and collectively reflected on their understanding of clinical reasoning in osteopathy.

### Focus groups

Three focus groups, each of 90 minutes duration, were conducted in a meeting room at SCU, at times convenient for all participants. A total of nine participants representing each participating institution (four from SCU, two from VU, two from Unitec and one from BSO) attended in person or by Skype. In Focus Group 1 four members from SCU met face to face with two representatives from VU. In Focus Group 2 all six attendees met with two representatives from Unitec, New Zealand via Skype. In Focus Group 3 the four members of SCU met with a representative of the BSO.

The facilitator used a semi-structured interview guide to explore two key questions:

- What is clinical reasoning in osteopathy?
- Is clinical reasoning in osteopathy different from clinical reasoning in other health disciplines?

The interview guide was adapted after each round of data collection and analysis so that leads from previous focus groups could be pursued as they arose, and so that progressive data collection could generate and refine emerging theory.

### Critical reflections

This study also drew on Brookfield’s [45] theory of critical self-reflection. Critical self-reflection encourages a deep evaluation of reasons, both obvious and not-so-obvious, for thinking and acting. The focus groups allowed ideas and concepts about clinical reasoning in osteopathy to be discussed, challenged, refined and developed within the group. After the focus groups, participants were asked to further reflect on their understandings of clinical reasoning in osteopathy and to write their own definition of clinical reasoning. When all written reflections had been submitted, they were collated by one member of the research team and forwarded to the other members for reading and reflection.

### Data analysis

Focus groups were audio recorded with participants’ consent and each one analysesd before the following stage of data collection. This process was continued until redundancy of information was reached. Transcriptions and written definitions were thematically analysed using the following procedure: Each member of the research team independently scrutinized the transcripts by repeatedly reading and re-reading to generate conceptual categories. Next, the whole research team met to compare conceptual codes and to develop inductive categories. Finally categories were compared and contrasted and searched for contradictions until agreement was reached. Theoretical sampling relied on the use of four education institutions in three continents, and a facilitator who provided a perspective from outside osteopathy.

## Results

Five key themes emerged from the data:

### Clinical reasoning does not lead to a single diagnosis

According to participants, the primary purpose of clinical reasoning in osteopathy was to develop a working diagnosis or rationale for treatment; practitioners were less concerned about naming a medical condition. As one participant said: *The aim isn’t to come up with a diagnosis but with a treatment plan (Participant S).* Another commented:

*In osteopathy you rarely come to a single label that conclusively says what this case is about … it’s imprecise. There’s no one answer like there is in a medical exam where the answer is infective endocarditis, for example. That single diagnosis is not there in osteopathy. (Participant P)*

In order to find ways of treating patients, practitioners synthesised findings from clinical histories, physical examinations and their own previous knowledge and experience. Clinical reasoning was used as a guide to the next phase of the consultation. Working diagnoses or hypotheses about patients’ conditions involved likely aetiologies for presenting signs and symptoms, and precipitating and maintaining factors. An example of an osteopathic working diagnosis might be: *peripheral inflammatory nociception of radiocarpal joints, caused by rheumatoid arthritis and maintained by occupational stress and sedentary lifestyle* (Participant R). Participants acknowledged the importance of palpatory findings in their working diagnoses: *I always find something, something in the tissues - the quadratus lumborum might be tight so I’ll work on it for a while (Participant M).* The working diagnosis for this patient was not a single, named medical condition.

### Clinical reasoning occurs in many contexts

According to participants, clinical reasoning occurs in many contexts including those of the patient and the practitioner. Adopting a patient-centred approach clearly influenced the clinical reasoning process:

*Typically [clinical reasoning] is collaborative – practitioner and patient, practitioner and patients’ families, practitioner and other practitioners (Practitioner U).*

Participants spoke of the importance of cultivating a patient-centred approach in their students. Students needed to understand that their own values and beliefs, past experience and evidence-based knowledge had to be mitigated by those of their patients.

[*We] encourage students to take a view of the patient that encompasses a whole range of attitudinal and affective components - not just facts-based or knowledge-based information on outcomes of clinical testing. While somebody may be experiencing significant lower back pain with high VAS [visual analogue pain scale] and limited ROM [range of motion] scores, it is the context that exists for the person in terms of their expectations of mobility, in terms of being able to get to the shops, look after two small children etc. - those things colour the value of that evidence in helping us establish what is important as an outcome for the patient. (Participant L)*

The process of clinical reasoning was described as interactive and dynamic. Consistent with a patient-centred approach, the reasoning process engaged the patient through a continual and evolving sequence of data gathering (including history taking and physical examination) and feedback from patients, their families and other health practitioners. One participant emphasised the terms ‘working diagnosis’ and ‘hypothesis’ and discouraged using ‘diagnosis’ to highlight the fluid nature of the clinical reasoning process.

The practitioners’ context was described as being strongly influenced by the challenges of practising a discipline that has a long tradition of anecdotal clinical efficacy but little scientific evidence specifically supporting osteopathic approaches. Where scientific evidence existed, it was used in the clinical reasoning process:

*You look at the evidence, you look at the quality of that evidence, the context within which it sits and the relationship to the decision you are making*. (Participant L)

Although osteopaths drew on evidence from other health disciplines (e.g. physiotherapy and chiropractic) that was relevant to their own practices, little research has been conducted on distinctly osteopathic approaches and treatments for a range of conditions. Participants’ comments included:

*We rely heavily on tacit knowledge and traditional knowledge. (Participant K)*

*You are left with very little evidence that guides you, and you have to reason from principles, past experience, case series and the patient context. This would then also include so-called collaborative reasoning where the patient’s previous experience, expectations and needs are brought into the equation. (Participant P)*

Clinical reasoning also drew on environmental influences beyond the patient’s and the practitioner’s immediate context. One participant pointed out that the knowledge that was used in clinical reasoning could be drawn from many sources:

*I think you draw on all knowledge when you are in clinic. It’s not just about ‘clinical knowledge’. I might have read a book or a patient might want to talk about something that comes from another realm, not a clinical realm*, *and these things influence your reasoning.* (Participant R)

### Clinical reasoning occurs in two different stages

Participants described two stages of clinical reasoning: the first involved an analysis of data to identify red flags for serious underlying pathology. Its purpose was specifically related to patient safety, namely, to identify patients requiring referral for medical or other health care. If patients were deemed suitable for osteopathic treatment, then osteopaths would attempt to clinically reason from a specifically osteopathic perspective.

*I think the first thing is the orthopaedic and neurological level to rule out any nasties, anything that looks like an organic disease, and then take a look at what’s happening in the physical body using all the osteopathic diagnostic techniques that give you information relative to the case. (Participant K)*

*I think there are two levels. The first is safety and I think in practice patient safety is reasonably assessed - cardinal signs, red flags. I think teachers and well-trained examiners know when to tick the patient safety box when they are assessing clinical reasoning. But that’s only a chunk of the reasoning. It’s not difficult. It’s much more ‘textbook’ to learn. The second level is osteopathic reasoning. The osteopathic focus. (Participant P)*

This osteopathic perspective referred to specific osteopathic diagnostic procedures, including diagnosis of soft tissue changes and diagnosis of restricted motion.

### Clinical reasoning calls on a number of metaskills

Participants acknowledged that clinical reasoning in osteopathy required a number of metaskills on the part of the practitioner, including knowledge generation when practitioners analysed and synthesised data to form working diagnoses:

*I see clinical reasoning as the process of gathering all relevant information including previous reports, imaging reports, test results, findings from physical examination etc., regarding a given patient, and processing it appropriately for the benefit of the patient.* (Participant R)

Reflexivity - when practitioners reflected on their own abilities and limitations and how their personal insights influenced their clinical reasoning - was widely acknowledged by participants as an essential component of the clinical reasoning process:

*Practitioners need to be able to reflect on action, but also importantly to reflect in action, that is, to analyse, synthesise, evaluate, and problem-solve many times as part of the normal business of practice. (Participant S)*

### Clinical reasoning in osteopathy is different from clinical reasoning in other health disciplines

Most participants stated that clinical reasoning in osteopathy was different from clinical reasoning in other health professions. It was acknowledged that the dual processes of hyptothetico-deductive reasoning and pattern recognition were at play in clinical reasoning in osteopathy as they appear to be in other health disciplines. However, according to participants, the difference was that clinical reasoning in osteopathy was guided by models that are grounded in osteopathic philosophy. These models were well known to the participants: not only had they been taught these models during their own pre-professional education, but also they were now involved in cultivating their use in the clinical reasoning processes of their students. These models are summarised in Table 2. Participants concurred with Participant U’s comment below. Many had similar experiences with students from other disciplines:

*We’ve taken physiotherapists into the masters’ course. Our tutors pick up that they have a very different way of thinking and it’s the clinical reasoning [in osteopathy] part that they struggle with the most. (Participant U)*

One participant described her perception of a discipline-specific approach to clinical reasoning this way:

*[The difference] has to be something to do with the osteopathic lens - the osteopathic way of looking at the world … (Participant S)*

Opinions were divided about the relationship between the clinical reasoning process and the osteopathic principles (the inter-relationship of body structure and function, the body’s inherent self-regulation, the importance of the somatic tissues in overall health). On the one hand, they were envisaged as *forming an overarching framework (Participant S)* that rendered an osteopathic approach to clinical reasoning different from that of other health practitioners. Another participant disagreed: *The principles are vexatious really and we make little reference to them. (Participant U)*

## Discussion

The purpose of this study was to understand clinical reasoning in osteopathy from the perspective of clinical educators and to determine the extent to which clinical reasoning in osteopathy was the same as, or different from, clinical reasoning in other health professions. Where commonalities exist, shared learning opportunities may be available, and osteopathic educators can confidently draw on scaffolded clinical reasoning exercises and assessment practices from other disciplines. Should further research confirm that clinical reasoning in osteopathy is distinct from other health professions, then clinical reasoning in osteopathy can contribute a different perspective to multidisciplinary decision-making and potentially enhance the quality of patient care.

### Similarities between clinical reasoning in osteopathy and other health professions

According to participants, the goal of clinical reasoning in osteopathy was not to reach a definitive medical diagnosis, but to identify and prioritise osteopathic treatment approaches. Clinical practice has been described as an encounter of considerable ambiguity [46] and perhaps even more so for osteopaths for whom a high proportion of their patients have chronic complex conditions for which medical diagnoses have not been found [47, 48]. Study participants used their clinical reasoning to direct their treatments based on osteopathic diagnosis, often in the absence of a named medical diagnosis. Research-informed practice calls on practitioners to consider all available evidence when formulating diagnoses and treatment plans [49]. This was achieved through a multi-stage reasoning process that usually began with a biomedical approach to identify red flags for serious underlying pathology, and culminated in specific osteopathic diagnostic techniques that included complex palpatory examination of all tissues of the body. This dependence on palpatory finding in the reasoning process has been established to varying degrees in other manual therapies, supported by clinical guidelines which argued for the use of these more subjective findings to guide appropriate management, even in the absence of definitive diagnosis of musculoskeletal conditions [50, 51]. However, this dependence on subjective palpatory findings is controversial. In fact, Jones and Rivett [37] argued against solely tissue-based reasoning in favour of management based on activity/participation for effective treatment while continuing to evaluate clinical impressions.

Much of the clinical reasoning literature supports health practitioners’ use of multiple reasoning strategies (e.g. hypothetico-deductive and pattern recognition; diagnostic or procedural, interactive, and conditional or predictive reasoning; narrative reasoning; ethical reasoning; collaborative reasoning) [31, 32]. Participants in this study did not describe reasoning strategies beyond two diagnostic models: one grounded in biomedical science knowledge, and the other in osteopathy-specific knowledge. This may be a reflection of an approach in osteopathic curriculum to emphasise primary care responsibilities (i.e. initial screening for red flag conditions). However, although not explicitly described, the interactive process whereby data is gathered, including patient’s response to treatment, is consistent with the collaborative reasoning model of occupational therapists described by Fleming and Mattingly [28].

Study participants described contexts that are similar to those described in other professions [24]. For example, participants were well aware of the patient’s context (i.e. the acknowledgement and value of patient’s personal health and illness experiences) and the practitioner’s context (i.e. the influence that their own backgrounds, beliefs and biases could exert of their reasoning). Collaborations among practitioners are also likely to be confined to those with other osteopaths rather than with other health professions. This may ensue from and/or be the reason for the way that osteopathy is practised: most osteopaths in Australia work in sole practices or group practices with other osteopaths [52]. Although there is some evidence that osteopaths do engage in referral networks with other health professionals [48, 53], collaborative reasoning with other health professionals was not reported in our study. For osteopathy to fully contribute to multidisciplinary health care, sociocultural approaches to patient care, including collaborations concerning patient diagnoses and treatment, may need to be implemented in future osteopathic curricula. It is also worthy of note that the contexts of wider social responsibility and the global community described by Higgs and Jones [24] were not evident in the description of osteopathic clinical reasoning that emerged in this study.

Metaskills like hypothesis generation and reflexivity that were identified by study participants are also well described in other professions. For example, Jensen et al. [10], comparing occupational therapy and physical therapy, identified reflection and moral agency as critical aspect of clinical reasoning in both disciplines. Reflective self-awareness was also described in physiotherapy by Jones et al. [32]. In fact, it has been argued that the ability to monitor and regulate cognitive processes appropriately is characteristic of expert performance in any profession [54].

### Differences between clinical reasoning in osteopathy and other health professions

Clinical reasoning is grounded in the learning, craft knowledge and intuition of that profession [25]. Thompson et al. [55] described clinical reasoning in osteopathy as a spectrum of approaches from technical rationality to professional artistry, and this continuum incorporates the three aspects of reasoning identified by Paterson [25]. Being both practitioners and clinical educators, the participants in the present study described clinical reasoning from both perspectives. For example, one participant commented:

*We assume there is a difference to find … As practitioners we know there is a difference. We have got evidence from our students going in as physios (physiotherapists) in the Masters. They have little skills in clinical reasoning* from an osteopathic perspective. (Participant M)

Participant M was able to bring perspectives from her own practice experience as well as her experience as a clinical educator to the discussion. In this case, her exposure to the clinical reasoning process of a student from a different discipline reinforced her own practice experience.

Participants were divided over how well osteopathic principles were applied in practice (*The principles are vexatious really and we make little reference to them. Participant U).* However, they all supported the idea of two phases of clinical reasoning in osteopathy, that is, using a biomedical approach to exclude red flag conditions, followed by reasoning through the lens of osteopathic structure-function models as part of the ‘craft knowledge’ of osteopathy. They argued that interpreting patient data through these structure-function relationships enabled practitioners to explore connections between seemingly unrelated things, for example, a headache with a dysfunctional breathing pattern or with restricted movement in one knee. How strongly other osteopaths support this two phase approach could be debated and will inevitability vary from practitioner to practitioner. There was general agreement among participants that the ‘osteopathic lens’ that was applied during the reasoning process, distinguished clinical reasoning in osteopathy from clinical reasoning in other professions. Whether a difference in fact exists, requires further exploration.

### Implications

A focus on aspects of clinical practice that overlap across health professions may facilitate multidisciplinary health care. Global workforce reforms are driving many professions to re-evaluate their traditional boundaries and scopes of practice to make way for cross-boundary health care, such as advanced nursing practice and prescribing rights for podiatrists. Collaborations among practitioners from different disciplines may need to overcome preciously guarded professional boundaries if the patients’ best interests are to be served. In fact, one of the trends in clinical reasoning identified by Higgs and Jones [24] was interdisciplinary reasoning that ‘transcends the boundaries of professional groups (with their diverse backgrounds) and includes patients as part of multidisciplinary teams’. Future studies could look for similarities in clinical reasoning across related professions (e.g. physiotherapy, chiropractic and osteopathy) and across those professions with whom osteopaths share referral networks to identify how each profession can collaborate for optimal patient care. This process may also confirm or disprove the existence of an osteopathy-specific reasoning process. Should a clinical reasoning process that is unique to osteopathy be identified, osteopaths would need to be play a greater role in multidisciplinary decision making to bring their unique perspectives to patients’ health care.

There are also important educational implications that derive from this study. Commonalities in clinical reasoning could be taught across disciplines and support the promotion of generalist training in some areas. Scaffolded learning activities to develop clinical reasoning and assessment rubrics could be shared across disciplines and marked by clinical educators of other health disciplines.

### Limitations

The findings of this study are context-dependent and not intended to be generalised to other people or settings. Participants were both clinical educators and practitioners and their understandings of osteopathic clinical reasoning provided a rich data set. The rigour of the research was ensured by long engagement with the texts and triangulation with other literature in the field. There would be value in conducting a study similar to the present one with osteopaths who have no experience in an educational institution, in order to gather more practice-based opinions and reduce the possible influence of institutional and theoretical objectives in graduate outcomes that may be present in educators’ opinions.

## Conclusion

Study participants posited that clinical reasoning in osteopathy differed from clinical reasoning in other professions in its two-phase approach: using a biomedical approach to rule out red flag conditions initially, and then using an ‘osteopathic lens’ through which to interpret data and to inform treatment. This ‘osteopathic lens’ referred to diagnostic reasoning models that are based on the relationship between structure and function in the human body and guide the selection of treatment approaches. Such discipline-specific approaches to clinical reasoning can contribute to our understanding of professional identity, and to our understanding of the contributions that individual professions bring to multidisciplinary health care.

According to participants, clinical reasoning in osteopathy is used to guide treatment rather than to identify a named medical condition. Contexts (e.g. the patient’s illness experiences, the practitioner’s experience and biases), and metaskills (e.g. hypothesis generation, reflective self-awareness) were similar to those identified in the clinical reasoning processes of other health professions. Further emphasis may need to be given to collaborative clinical reasoning in osteopathy education and practice to ensure the best outcomes for patients.
